# Mitochondrial respiration in peripheral blood mononuclear cells correlates with depressive subsymptoms and severity of major depression

**DOI:** 10.1038/tp.2014.44

**Published:** 2014-06-10

**Authors:** A Karabatsiakis, C Böck, J Salinas-Manrique, S Kolassa, E Calzia, D E Dietrich, I-T Kolassa

**Affiliations:** 1 Department of Clinical & Biological Psychology, Institute of Psychology and Education, University of Ulm, Ulm, Germany; 2 AMEOS Clinic for Psychiatry and Psychotherapy Hildesheim, Hildesheim, Germany; 3 SAP Switzerland AG, Tägerwilen, Switzerland; 4 Department of Anesthesia, Section of Anesthesiological Pathophysiology and Process Development, University of Ulm, Ulm, Germany; 5 Burghof-Klinik, Rinteln, Germany

## Abstract

Mitochondrial dysfunction might have a central role in the pathophysiology of depression. Phenotypically, depression is characterized by lack of energy, concentration problems and fatigue. These symptoms might be partially explained by reduced availability of adenosine triphosphate (ATP) as a consequence of impaired mitochondrial functioning. This study investigated mitochondrial respiration in peripheral blood mononuclear cells (PBMCs), an established model to investigate the pathophysiology of depression. Mitochondrial respiration was assessed in intact PBMCs in 22 individuals with a diagnosis of major depression (MD) compared with 22 healthy age-matched controls using high-resolution respirometry. Individuals with MD showed significantly impaired mitochondrial functioning: routine and uncoupled respiration as well as spare respiratory capacity, coupling efficiency and ATP turnover-related respiration were significantly lower in the MD compared with the control group. Furthermore, mitochondrial respiration was significantly negatively correlated with the severity of depressive symptoms, in particular, with loss of energy, difficulties concentrating and fatigue. The results suggest that mitochondrial dysfunction contributes to the biomolecular pathophysiology of depressive symptoms. The decreased immune capability observed in MD leading to a higher risk of comorbidities could be attributable to impaired energy supply due to mitochondrial dysfunction. Thus mitochondrial respiration in PBMCs and its functional consequences might be an interesting target for new therapeutical approaches in the treatment of MD and immune-related comorbidities.

## Introduction

### The potential role of mitochondria in depression

Mitochondria produce adenosine triphosphate (ATP)—the main source of cellular energy. Energy that is liberated during the degradation of dietary intermediates is conserved via the mitochondrial respiratory chain as the so-called proton motive force across the inner mitochondrial membrane. The enzyme *ATP synthase* utilizes the energy conserved as the proton motive force for the conversion of adenosine diphosphate and inorganic phosphate to ATP. In addition, mitochondria are also pivotally involved in cellular calcium homeostasis,^1^ redox signalling,^2^ regulation of free radicals^3^ and the prevention or facilitation of apoptosis.^4^

Recently, mitochondrial dysfunction has been suggested to have an important role in the pathophysiology of major depression (MD).^5^ Characteristic depressive symptoms, for example, physiological (sleeping disturbances), psychological (lack of motivation) and neurocognitive alterations (lack of concentration, working memory deficits),^6^ might be explained on the one hand by pro-inflammatory states and sickness behaviour, which are commonly observed in depression,^7^ and on the other hand by alterations in energy metabolism and availability of ATP.

Furthermore, mitochondria are the main producers of reactive oxygen species. Several psychopathologies, including MD, have been reported to be associated with disturbed oxidative homeostasis.^8^ Positive correlations between the concentration of oxidative stress markers in peripheral blood and the chronicity as well as the severity of depression have been observed.^9, 10, 11^ Consequently, an increasing body of literature implicates reactive oxygen species as a major contributing factor to the physiological manifestations of psychopathologies.^12,13^ Defects in the mitochondrial oxidative phosphorylation system, in particular changes to the respiratory chain, are suggested to be involved in the pathogenesis of numerous neuropsychiatric disorders including MD.^13, 14, 15, 16, 17^

### Impaired energy metabolism in MD

Animal studies investigating the mitochondrial activity in chronically stressed rats, an animal model of depression, found that the activity of several complexes of the mitochondrial respiratory chain were reduced in the cortex of the animals.^14,18^ Neuroimaging studies in humans using positron emission tomography and single-photon emission computed tomography assessed metabolic rates in different brain regions, including prefrontal cortex and basal ganglia, revealing a low cerebral bioenergetic metabolism in MD patients.^19, 20, 21, 22^ Further, Gardner *et al.*^23^ showed that muscle mitochondria of depressed patients who presented concomitant physical symptoms produced less ATP and showed decreased ratios of different enzyme activities of the respiratory chain. The most prominent ratios were between *NADH-cytochrome C reductase* and *cytrochrome C oxidase* and between *succinate-cytochrome C reductase* and *cytochrome C oxidase*.^23^ These findings underline the observation that the biological consequences of MD are not limited to the brain, but spread out into the periphery.

Blunted immunity has been reported in psychiatric disorders including depression,^24, 25, 26^ posttraumatic stress disorder^27^ and schizophrenia,^28,29^ possibly leading to the higher risk for age-related comorbidities, for example, cardiovascular diseases,^30, 31, 32, 33^ type 2 diabetes^30,34^ and cancer.^35^ As cellular activity depends on mitochondrial energy supply, impaired mitochondrial capability of immune cells in depression results in impaired immunity^36^ and therefore offers a linkage to these observations.

First evidence for the possible consequences of depression on cells linked to immunity has been shown recently by Hroudová *et al.*,^37^ who found impaired mitochondrial respiration in thrombocytes of depressed individuals.

This study investigates mitochondrial respiration of peripheral blood mononuclear cells (PBMCs) in individuals with a diagnosis of MD compared with healthy age-matched controls. PBMCs, which encompass the main mediators of cellular immunity, are a common model for the investigation of the pathophysiological consequences of depression. We hypothesized that individuals with MD show a reduced mitochondrial activity compared with controls and that mitochondrial activity is negatively correlated with depressive symptom severity.

## Materials and methods

### Participants

To exclude possible gender-specific differences on respiratory activity, exclusively women were recruited. Forty-four female participants (age range 50–69 years, mean age = 58.2±5.8 years) were investigated in the study: 22 individuals with a current diagnosis of MD according to DSM-IV^38^ and 22 healthy age-matched controls. Individuals with a diagnosis of MD were recruited at the AMEOS Clinic for Psychiatry and Psychotherapy in Hildesheim, Germany. The controls were recruited by local public poster advertisement. The study was approved by the Ethics Committee of the Hanover Medical School. All participants gave written informed consent before participation. Control subjects received 40  € as compensation for participating in the study.

Exclusion criteria comprised presence of neuropsychiatric comorbidities including Parkinson's disease, Alzheimer's disease, schizophrenia and other clinically relevant neurologic or psychiatric disorders, anaemia, presence of severe immune alterations, autoimmune diseases and cancer. Subjects under medication with known effects on the immune system, for example, immunosuppressors, cytostatic agents and recent vaccines, were also excluded. Female age-matched controls without any depressive episodes in the past and without any depressive episodes within the last two consanguineous generations were recruited. The Beck Depression Inventory II (BDI-II, self-rating^39^) as well as the Montgomery-Asberg Depression Rating Scale (MADRS, interview^40^) were used to assess depression severity. The Essener Trauma Inventory (ETI, self-rating^41^) was used to assess traumatic events and to identify posttraumatic stress disorder. Three of the included individuals suffering from depression were diagnosed with concurrent posttraumatic stress disorder. In addition, the body mass index (BMI), the current smoking status and the status of physical activity were assessed for all study participants.

As one would expect, the depressed group showed significantly higher depressive symptoms in the BDI and the MADRS, and reported more traumatic events in the ETI than the control group (Table 1). Groups did not differ with respect to age and physical activity. The MD group showed, however, a significantly higher BMI and included significantly more smokers.

Although the control group was free of antidepressant and antipsychotic medication, 77.3% of the MD group took antidepressant medication (selective serotonin reuptake inhibitors (n = 5), serotonin–norepinephrine reuptake inhibitors (n = 3), tetracyclic antidepressants (n = 3), norepinephrine–dopamine reuptake inhibitors ( n= 1), tricyclic antidepressants (n = 1) and a combination of two different antidepressants (n = 4)) and 40.9% took antipsychotic medication. All individuals suffering from MD were taking other medications (including NSAIDs, antihypertensive drugs, levothyroxine), whereas 59.1% of the control group were free of general medication.

### Isolation and preparation of PBMCs for high-resolution respirometry

Peripheral blood (35  ml) was collected by venous puncture into EDTA-buffered collection tubes (Sarstedt, Nümbrecht, Germany). A Fiquoll-hypaque gradient was used to isolate PBMCs following the manufacturer's protocol (GE Healthcare, Chalfont St Giles, UK). Isolated PBMCs were stored at −80 °C in standard cryoprotective freezing medium (dimethyl sulphoxide: Sigma-Aldrich, St. Louis, MO, USA; fetal calf serum: Sigma-Aldrich; dilution: 1:10) until analysis. Frozen PBMCs were thawed, counted and resuspended in 6  ml mitochondrial respiration medium containing 0.5  mM ethylene glycol tetraacetic acid (Sigma-Aldrich), 3  mM MgCl_2_ (Scharlau, Sentmenat, Spain), 20  mM Taurine (Sigma-Aldrich), 10  mM KH_2_PO_4_ (Sigma-Aldrich), 20 mM HEPES (Sigma-Aldrich), 110  mM D-sucrose (Sigma-Aldrich), 1 g l^−1^ essentially fatty acid-free bovine serum albumin (Sigma-Aldrich), 60  mM K-lactobionic acid (Sigma-Aldrich) and 10  mM pyruvate (Sigma-Aldrich). A total volume of 2 ml of PBMC suspension was transferred into each oxygraph chamber and respiration rates (oxygen flux, J_O2_) were assessed by polarographic oxygen sensors over time as the rate of oxygen consumption by the biological sample. To exclude possible influences of the procedure on mitochondrial activity, a comparison of fresh and frozen PBMC samples was performed, which revealed no differences in the results. Bioenergetic analysis of mitochondrial respiration was performed in duplicates for each sample. Averaged values were used for statistical analysis.

### Characterization of mitochondrial activity with high-resolution respirometry

Activity of the mitochondrial respiration in PBMCs was measured using a high-resolution respirometer (Oxygraph-2k; Oroboros Instruments, Innsbruck, Austria) set to 37  °C with a stirring speed of 750  r.p.m. Data recording was performed using the DatLab software 5.1.0.20 (Oroboros Instruments) with a sampling rate of 0.5  Hz. Manual titration of inhibitors and uncouplers was performed using Hamilton syringes (Hamilton Company, Reno, NV, USA). For the characterization of mitochondrial activity the following characteristics were assessed: (1) Physiological respiratory activity in intact cells (routine respiration), (2) *LEAK* respiration (residual respiration after inhibition of ATP synthesis compensating for the proton leak, proton slippage and cation cycling across the inner mitochondrial membrane), (3) maximal capacity of the respiratory chain (nonphysiological maximal uncoupled respiration, that is not limited by the enzyme activity of the *ATP synthase*) and (4) residual oxygen consumption (respiration attributable to other cellular oxygen-consuming processes besides the respiratory chain). At first, routine respiration (no additives, R) was measured, *LEAK* respiration (L) was induced by the addition of 0.5  μl *ATP synthase* inhibitor oligomycin (final concentration 5  μM; Sigma-Aldrich), and maximal capacity of the respiratory chain was determined after uncoupling by stepwise titration (usually 1–2 steps) of 1  μl FCCP (final concentration 0.5 μM; Sigma-Aldrich) until maximal uncoupled respiration was measured. Residual oxygen consumption was determined after sequential addition of 5  μl complex I-inhibitor Rotenone (final concentration 0.5  μM; Sigma-Aldrich) and 1  μl complex III-inhibitor antimycin A (final concentration 2.5  μM; Sigma-Aldrich), which subtracted from the other parameters. Absolute respiration values were normalized for the total number of cells per chamber. Respiration related to ATP turnover was calculated as the difference between routine and *LEAK* respiration (R−L) and spare respiratory capacity as the difference between maximal uncoupled respiration and routine respiration (E−R).^42^

Following the recommendations of the manufacturer (Oroboros Instruments), the following flux control ratios were calculated for further analyses: routine flux control ratio (routine respiration over uncoupled respiration, R/E) and coupling efficiency (ATP turnover over routine respiration, (R−L)/R).^42,43^ Determination of flux control ratios offers the advantage of an internal normalization for an evaluation of respiratory parameters independent of cell size and mitochondrial content. As the routine flux control ratio is normalized to the maximal capacity of the respiratory chain, it represents the efficiency of mitochondrial respiration.

Due to technical reasons six samples were included into the evaluation of routine respiration, but had to be excluded from the analysis of oxygen flux following the induction of *LEAK* respiration.

### *Citrate synthase* activity

The activity of the enzyme *citrate synthase* can be used as a quantitative measure for the determination of intracellular density of mitochondria. Following respirometry, cells were removed from the chamber and an aliquot of 1 ml was given into liquid nitrogen for the preparation of the *citrate synthase* activity assay. The activity of the enzyme was determined spectrophotometrically at 30  °C as previously described.^44^ The assessment of *citrate synthase* activity was performed in duplicates for each sample and averaged values were used for statistical analysis. Values were corrected to a given *citrate synthase* standard.

### Statistical analysis

The data were statistically analysed with SPSS (version 20; IBM, Armonk, New York, NY, USA) and R 3.0.2^45^ on a Windows platform (Microsoft, Redmond, WA, USA). Before analysis, residuals were tested for normal distribution (Shapiro–Wilk test^46^) and equality of variance (Levene's test^47^). Nonparametric tests were used where appropriate. Group comparison of descriptive and clinical variables and mitochondrial respirometric parameters were calculated using Student's independent *t*-test (parametric),^48^ Mann–Whitney *U* test (nonparametric)^49^ or Pearson's *X*^2^ test^50^ for categorical variables. According to the underlying hypotheses either a one-tailed or a two-tailed test was performed. The significance level was set to *P*<0.05. Values are given as means±standard deviations (s.d.).

To test the hypothesis that the severity of depression correlates negatively with mitochondrial oxygen flux, Kendall-*τ*-b correlation modelling^51^ was performed for the respective respirometric parameters with the BDI as well as the MADRS as measures for depression severity. Sub-items were selected that represent physiological traits associated with a reduced energy status, including ‘loss of energy', ‘fatigue' and ‘inactivity', as well as ‘difficulties concentrating' and ‘disturbed sleep pattern'. Moreover, sub-items characterizing psychological traits were also tested, including ‘sadness', ‘loss of interest' and ‘irritability'. In addition, we correlated mitochondrial oxygen flux with traumatic load, as indicated by the ETI.

## Results

### Mitochondrial respiration of PBMCs is significantly decreased in MD patients

**Figure 1:**
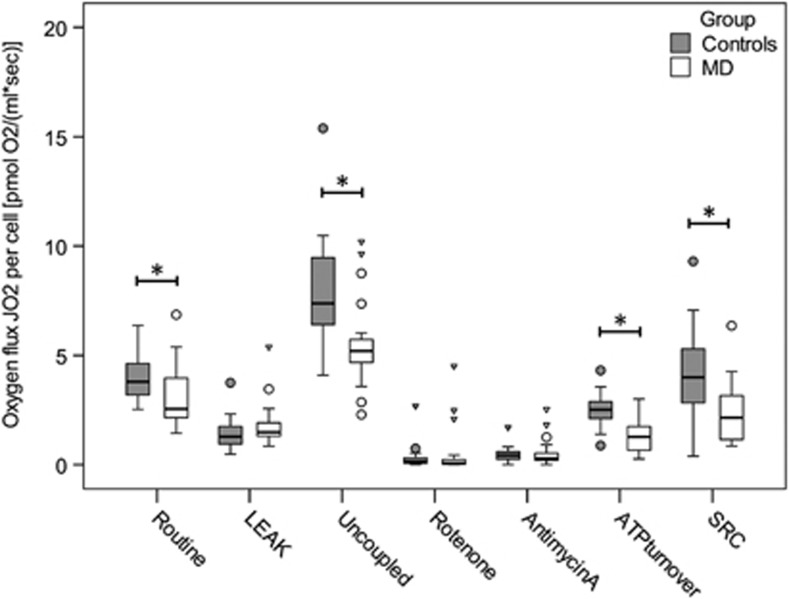
Boxplots of group-wise comparison of respiration in PBMCs from MD patients (*n* = 19) and control subjects (*n*  = 19) characterized by mitochondrial oxygen flux (J_O2_) per cell. Circles and triangles represent statistical outliers of the respective groups. Asterisk (*) indicates significant group differences on an alpha level of 0.05 (one-tailed Mann–Whitney *U* test or Student's independent *t*-test). MD, major depression; PBMCs, peripheral blood mononuclear cells; SRC, spare respiratory capacity.

PBMCs of the MD group showed a significantly lower routine respiration and maximal uncoupled respiration compared with the control group (Table 2,  Figure 1). Furthermore, individuals with MD showed a significant reduction of ATP turnover-related respiration and spare respiratory capacity. No differences were observed between both groups with respect to *LEAK* respiration and residual oxygen consumption. Exclusion of the patients diagnosed with a comorbid posttraumatic stress disorder did not alter the result pattern. Determination of *citrate synthase* activity revealed significantly higher levels for the MD group compared with the control group (MD: 4.7±4.3  nmol min^−1^ per 10^6^ cells, C: 2.9±1.5  nmol min^−1^ per 10^6^ cells, *U= 130.5, Z*= −2.442, *P*= 0.015).

**Figure 2:**
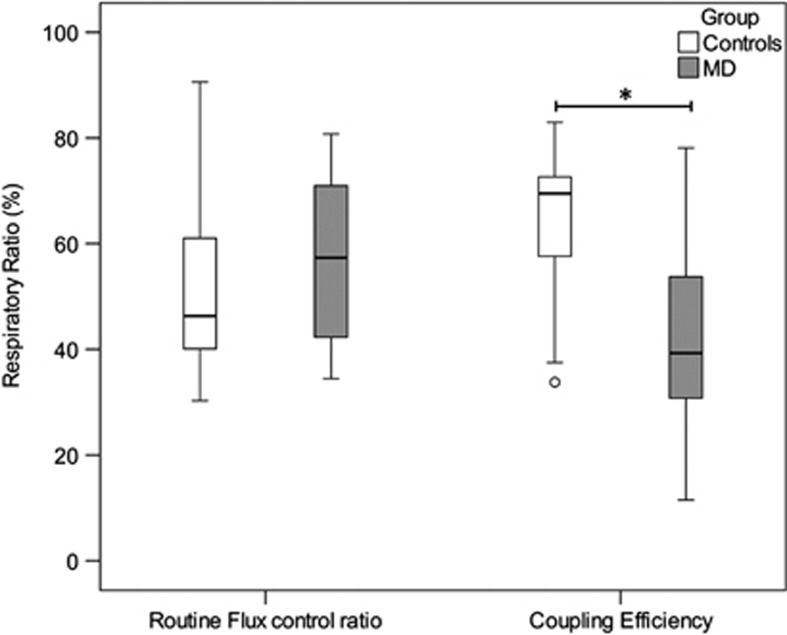
Boxplots of group-wise comparison of respiration in PBMCs from MD patients (*n* = 19) and control subjects (*n* = 19) characterized by flux control ratios. Circles and triangles represent statistical outliers of the respective groups. Asterisk (*) indicates significant group differences on an alpha level of 0.05 (one-tailed Mann–Whitney *U* test or Student's independent *t*-test, respectively). MD, major depression; PBMCs, peripheral blood mononuclear cells.

Internal normalization through calculation of flux control ratios revealed a significant reduction of coupling efficiency for PBMCs of MD patients (Table 2,  Figure 2). Routine flux control ratio showed no significant difference between the two groups.

### Decline of respiratory activity correlates with depressive symptom severity

**Figure 3:**
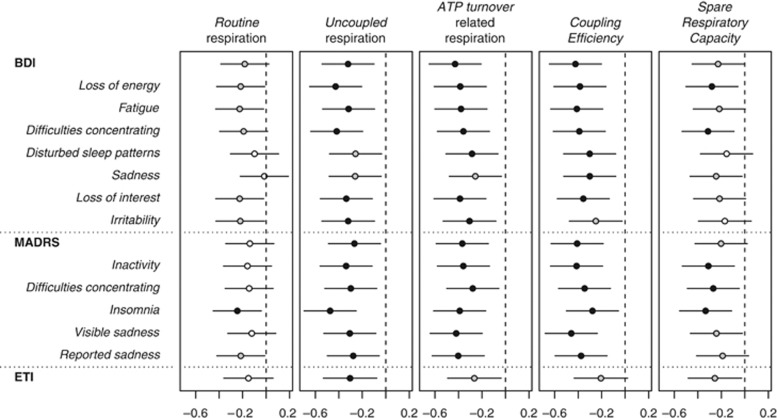
Graphical illustration of the correlation analysis between mitochondrial respiration in PBMCs and depressive symptom severity (BDI, MADRS; sum score and selected subscales) as well as ‘traumatic load' (ETI). The horizontal scale shows Kendall's *τ* rank correlation coefficient, dashed vertical lines indicate absence of association (*τ* = 0). Respective *τ* coefficients are given as circles with 95% confidence intervals as horizontal bars. Black circles mark a correlation with a significance of *P<*0.01, grey circles mark a significance of 0.01⩾*P*<0.05 and white circles mark nonsignificant correlations (one-tailed Kendall-*τ*-b correlation analysis). ATP, adenosine triphosphate; BDI, Beck Depression Inventory II; ETI, Essener Trauma Inventory; MADRS, Montgomery-Asberg Depression Rating Scale; PBMCs, peripheral blood mononuclear cells.

As shown in Figure 3, mitochondrial respiration correlated negatively with depressive symptom severity as measured by BDI and MADRS. This also held true with selected subscales of the two questionnaires, including physiological symptom clusters as ‘loss of energy', ‘fatigue', ‘difficulties concentrating', ‘inactivity' and ‘disturbed sleep patterns/insomnia' as well as other depressive symptoms such as ‘sadness', ‘loss of interest' and ‘irritability'. Moreover, mitochondrial respiration correlated negatively with ‘traumatic load' as assessed with the ETI (see also Supplementary Table S1).

## Discussion

This study shows that the rate of mitochondrial respiration in PBMCs of acutely depressed individuals is reduced compared with healthy controls. The significant reduction of routine respiration, ATP turnover-related respiration and coupling efficiency suggests a lower ATP availability at basal state as well as a reduced efficiency of ATP production in immune cells.

The changes of mitochondrial oxygen flux observed in PBMCs of depressed patients were not attributable to a lower density of mitochondria per cell as confirmed by the analysis of *citrate synthase* activity. The findings indicate that mitochondrial density is higher in PBMCs of MD patients. Furthermore, the significant decrease of maximal uncoupled respiration and spare respiratory capacity indicates that cells of acutely depressed patients have a reduced capacity of mitochondrial respiration. This decreased capacity might result in a lack of energy in times of higher energy demand as for example under stress conditions and inflammation.^36^

On a functional level the decrease of routine respiration and ATP turnover-related respiration could be either due to a lower activity of the mitochondrial respiratory chain or due to changes in substrate supply and availability of coenzymes. As the routine control ratio, which evaluates the efficiency of mitochondrial respiration, was unchanged in acutely depressed patients compared with controls, we assume that the changes observed in PBMCs might originate rather from a reduced capacity of the respiratory chain. This interpretation is in agreement with the findings of Hroudová *et al.,*^37^ who showed in a comparison of intact versus permeabilized blood platelets that the measured decrease in physiological respiration is at least partially caused by alterations in substrate supply and availability of coenzymes.

Evaluation of residual oxygen consumption demonstrated no significant alterations; this indicates that the changes observed in mitochondrial oxygen flux in PBMCs of acutely depressed patients are not attributable to alterations of other cellular oxygen-consuming processes, but to changes of the respiratory chain.

Higher depressive symptom scores in the BDI and MADRS (sum scores and subscales) correlated negatively with mitochondrial activity. The correlations were not limited to the subscales characterizing energy status of the affected individuals, for example, ‘lack of energy', ‘fatigue' or ‘difficulties concentrating', but were also found for other depressive symptoms such as ‘sadness' and ‘irritability' and ‘traumatic load' as assessed with the ETI. These results show that mitochondrial activity is linked correlatively to a series of both physiological and psychological characteristics of depression and they suggest that mitochondrial dysfunction contributes to the biomolecular pathophysiology of depressive symptoms, possibly by sickness behaviour and increased inflammatory states—a prominent finding in MD.^7^ The molecular pathways underlying these observations need further investigation. A disturbance of the mitochondrial energy supply, either as a cause or consequence of depression, could be one factor for the impaired functioning of the immune system in depression and the higher incidence of age-related comorbidities.

Furthermore, mitochondrial activity measured in PBMCs appears to be a promising new candidate for a ‘biomarker' of depression, which could provide important clinical information, for example, about the effectiveness of therapeutic interventions and, in particular, the response to antidepressant treatment. It needs further investigation whether the response to antidepressant medication could be tested *in vitro* before the intake to increase treatment efficacy. For these purposes, it should be assessed in a longitudinal study design how mitochondrial respiration changes with the status of depression.

### Limitations and outlook on future studies

One limitation of this study is that the groups differed in medication, as individuals with MD took antidepressant medication. To date we do not know whether different antidepressants have differential effects on mitochondrial function. Hroudová and Fisar^52^ showed that several antidepressants especially inhibited the activity of the respiratory complexes I and IV in isolated pig brain mitochondria, whereas Scaini *et al.*^53^ showed that chronic administration of diverse antidepressants to rats led to an increased activity of the respiratory complexes I, II and IV in different brain regions. However, given the hypothesis that mitochondrial activity is reduced during depressive states, which is underlined by the findings of this study, antidepressant medication should rather act as an enhancer of mitochondrial function for an effective treatment of depression than further inhibit mitochondrial activity. If the changes of mitochondrial respiration observed in this study are indeed attributable to antidepressant medication, the question arises whether these changes are side effects or actually contribute to the therapeutical mechanism of action. Further studies are needed including drug-naive depressed patients, at best investigating the mitochondrial respiratory profile before and after treatment with antidepressant medication.

Another limitation of this study is that it cannot be ruled out that the significant difference in smoking status and BMI between the two groups may have influenced the results. Fariello *et al.*^54^ showed for sperm cells a positive correlation of a higher BMI with reduced mitochondrial activity. Furthermore, it was shown that smoking acutely inhibits complex IV of the respiratory chain in PBMCs.^55^ However, as it is well known that depression is associated with a higher BMI as well as an increased probability of smoking,^56^ the exclusion of smokers as well as the exclusion of individuals with a higher BMI would have led to a nonrepresentative group of depressed individuals. This is the typical problem of covariance analysis with groups that inherently differ in one particular characteristic.^57^

## Conclusions

In summary, our findings of a decreased mitochondrial respiratory activity in PBMCs of depressed patients support the hypothesis that mitochondria have an essential role in the blunted immunity in depression and might contribute to the pathophysiology and/or aetiology of depressive symptoms. The precise character and the associated molecular mechanisms underlying this relationship remain to be elucidated. Mitochondrial activity was negatively correlated with the severity of depression and could account for distinct features of the depressive phenotype. Pharmacotherapeutical treatment options targeting mitochondrial alterations in the setting of depression might improve depressive symptoms and might prepare patients for cognitive behavioural therapy by ameliorating severe states of lack of energy. Future studies should investigate whether successful treatment normalizes mitochondrial function and whether mitochondrial respiratory activity in PBMCs could be used as a state (bio)marker of depression.

## Figures and Tables

**Table 1:** Mean and s.d. of clinical characteristics, in *n*  = 22 MD patients and *n*=22 control subjects

*Variables*	*Groups*		U*-value*	Z*-value*	*Х*^*2*^	P*-value*
	*MD (*n*=* *22)*	*Controls (*n*=* *22)*				
*DSM-IV*						
Mild	4 (18.2%)	0				
Moderate	8 (36.4%)	0				
Severe	10 (45.5%)	0				
						
BDI score (mean±s.d.)	24.0±10.4	2.3±2.3	0.5	−5.683		**1.32** × **10**^−^^**8**^
MADRS score (mean±s.d.)	23.4±8.7	2.0±2.3	0.5	−5.706		**1.16** × **10**^−**8**^
ETI score (mean±s.d.)	28.0±17.3	6.6±7.0	48.0	−4.339		**1.40** × **10**^−**5**^
Age (mean±s.d. years)	58.7±6.2	57.6±5.6	219.0	−0.541		0.588
Sex (female, n (%))	22 (100%)	22 (100%)				
BMI (mean±s.d. kg m^−^^2^)	28.5±7.2	24.4±3.0	155.0	−2.042		** 0.041**
Smoker (yes, n (%))	12 (54.5%)	4 (18.2%)			6.29	** 0.012**
Physical activity (yes, n (%))	14 (63.6%)	19 (86.4%)			3.03	0.082
						
*Medication*						
Medication (yes, n (%))	22 (100 %)	9 (40.9 %)				
Antidepressant (yes, n (%))	17 (77.3 %)	0				
Antipsychotic (yes, n (%))	9 (40.9 %)	0				

Abbreviations: BDI, Beck Depression Inventory II; BMI, body mass index; ETI, Essener Trauma Inventory; MADRS, Montgomery-Asberg Depression Rating Scale; MD, major depression. *U*-value, *Z*-value are listed for two-tailed Mann–Whitney *U* test, *X*^2^ for Pearson's *X*^2^ test. Bold *P*-values indicate significance on an alpha level of 0.05.

**Table 2:** Respiratory parameters assessed in PBMCs of MD patients and control subjects

*Variables*	*Groups*		T*-value*	U*-value*	Z*-value*	*df*	P*-value*
	*MD (*n *=* *19)*	*Controls (*n *=* *19)*					
Routine respiration (R)^a^	3.35±1.44	4.03±1.04	−1.80			38.3	**0.040**
*LEAK* respiration (L)	1.83±1.06	1.47±0.76		133.5	−1.372		0.085
Uncoupled respiration (E)	5.55±2.10	8.04±2.57		68.0	−3.284		**5.11** × **10**^−^^**4**^
							
*Residual oxygen consumption (ROX)*							
Rotenone	0.55±1.2	0.33±0.60		135.0	−1.330		0.092
Antimycin A	0.54±0.66	0.45±0.39		162.0	−0.541		0.294
							
ATP turnover-related respiration (R−L)	1.34±0.81	2.54±0.78	−4.64			36	**2.25** × **10**^−^^**5**^
Spare respiratory capacity (E−R)	2.38±1.41	4.03±2.26		98.0	−2.409		**0.008**
Routine flux control ratio (R/E)	58.00±15.69	53.00±18.24		146.0	−1.007		0.157
Coupling efficiency ((R−L)/R)	41.18±15.71	63.83±13.76	−4.73			36	**1.70** × **10**^−^^**5**^

Abbreviations: MD, major depression; PBMCs, peripheral blood mononuclear cells.

Values are given as mean±s.d. pmol O_2_ per s·ml for the upper part and mean±s.d. % for the lower part.

a Routine respiration was assessed in *n* = 22 MD patients and *n* = 22 control subjects. *U*-value, *Z*-value are listed for one-tailed Mann–Whitney *U* test, *T-*value and df are listed for one-tailed Student's independent *t-test*. Parametric and nonparametric tests were applied where appropriate. Bold *P*-values indicate significance on an alpha level of 0.05.
