# Supplementary material for: Mitochondrial respiration in peripheral blood mononuclear cells correlates with depressive subsymptoms and severity of major depression
Source: Transl Psychiatry. 2014 Jun 10;4(6):e397–. doi: 10.1038/tp.2014.44 (PMC4080325; doi:10.1038/tp.2014.44)
Supplement: Supplementary Table S1 [file tp201444x1.doc]

Table S1 shows the values of the one-tailed *Kendall-τ-b* correlation analysis between mitochondrial respiration in PBMC and depressive symptom severity (BDI, MADRS; sum score and selected subscales) as well as “traumatic load” (ETI).

**Supplementary Table S1. *Correlation Analyses of Respiratory Parameters with the Severity of Depression and Traumatic Load.***

| **Questionnaire** | | | | **Respirometric parameters** | | | | | | | | | | | |  | | | |  | | |
| --- | --- | --- | --- | --- | --- | --- | --- | --- | --- | --- | --- | --- | --- | --- | --- | --- | --- | --- | --- | --- | --- | --- |
|  | | | | ***Routine* respiration** | | | ***Uncoupled* respiration** | | | ***ATP turnover***  **related respiration** | | | ***Coupling Efficiency*** | | | ***Spare Respiratory Capacity*** | | | |  | | |
| **Items** | | | | *τ* | | *P-value* | *τ* | *P-value* | | *τ* | *P-value* | | *τ* | | *P-value* | *τ* | | *P-value* | |  | | |
|  | | | |  | | |  | | |  | | |  | | |  | |  | | | | |
| **BDI** | | | | - .181 | | **.041** | - .321 | **.002** | | - .429 | **7.48 × 10^-5^** | | - .423 | | **9.20 × 10^-5^** | - .228 | **.022** | | | |  | |
|  | *Loss of energy* | | | | - .217 | **.019** | - .427 | | **7.97 × 10^-5^** | - .385 | **3.32 × 10^-4^** | - .385 | | **3.32 × 10^-4^** | | - .280 | **.007** | |  | | | |
|  | *Fatigue* | | | | - .225 | **.016** | - .317 | | **.003** | - .379 | **4.08 × 10^-4^** | - .412 | | **1.37 × 10^-4^** | | - .218 | **.027** | |  | | | |
|  | *Difficulties concentrating* | | | | - .191 | **.034** | - .418 | | **1.10 × 10^-4^** | - .359 | **7.65 × 10^-4^** | - .309 | | **2.83 × 10^-4^** | | - .313 | **.003** | |  | | | |
|  | *Disturbed sleep pattern* | | | | - .098 | .174 | - .258 | | **.011** | - .285 | **.006** | - .302 | | **.004** | | - .155 | .085 | |  | | | |
|  | *Sadness* | | | | - .015 | .443 | - .260 | | **.011** | - .256 | **.012** | - .301 | | **.004** | | - .244 | **.015** | |  | | | |
|  | *Loss of interest* | | | | - .225 | **.016** | - .336 | | **.001** | - .387 | **3.11 × 10^-4^** | - .356 | | **8.29 × 10^-4^** | | - .215 | **.029** | |  | | | |
|  | *Irritability* | | | | - .220 | **.019** | - .320 | | **.003** | - .306 | **.004** | - .250 | | **.015** | | - .171 | .069 | |  | | | |
|  | |  |  |  | |  |  |  | |  |  | |  | |  |  |  | | | |  | |
| **MADRS** | | | | - .139 | | .092 | - .267 | **.009** | | - .368 | **5.81 × 10^-4^** | | - .409 | | **1.51 × 10^-4^** | - .202 | | **.037** | | | |  |
|  | *Inactivity* | | | | - .159 | .064 | - .338 | | **.001** | - .359 | **7.62 × 10^-4^** | - .414 | | **1.28 × 10^-4^** | | - .311 | **.003** | | | |  | |
|  | *Difficulties concentrating* | | | | - .144 | .084 | - .298 | | **.004** | - .278 | **.007** | - .345 | | **.001** | | - .269 | **.009** | | | |  | |
|  | *Insomnia* | | | | - .245 | **.009** | - .474 | | **1.39 × 10^-5^** | - .389 | **2.91 × 10^-4^** | - .278 | | **.007** | | - .334 | **.002** | | | |  | |
|  | *Visible sadness* | | | | - .122 | .122 | - .306 | | **.003** | - .420 | **1.04 × 10^-4^** | - .459 | | **2.52 × 10^-5^** | | - .242 | **.016** | | | |  | |
|  | *Reported sadness* | | | | - .216 | **.019** | - .277 | | **.007** | - .402 | **1.89 × 10^-4^** | - .375 | | **4.57 × 10^-4^** | | - .189 | **.047** | | | |  | |
|  | |  | |  | |  |  |  | |  |  | |  | |  |  | |  | | | |  |
|  | |  |  |  | |  |  |  | |  |  | |  | |  |  | |  | |  | | |
| **ETI** | |  |  | - .150 | | .081 | - .304 | **.005** | | - .265 | **.011** | | - .207 | | **.038** | - .255 | | **.014** | | | | |
|  | | | |  | |  |  |  | |  |  | |  | |  |  | |  | | | | |

Abbreviations: BDI, *Beck Depression Inventory II*; MADRS, *Montgomery-Asberg Depression Rating Scale*; ETI, *Essener Trauma Inventory*. Correlation coefficients (*τ*) and *P-values* were determined using one-tailed *Kendall-τ-b correlation* modelling. Bold *P-values* indicate significance on an alpha level of 0.05.
